# Temporal variations of ^90^Sr and ^137^Cs in atmospheric depositions after the Fukushima Daiichi Nuclear Power Plant accident with long-term observations

**DOI:** 10.1038/s41598-020-78312-3

**Published:** 2020-12-10

**Authors:** Takeshi Kinase, Kouji Adachi, Tsuyoshi Thomas Sekiyama, Mizuo Kajino, Yuji Zaizen, Yasuhito Igarashi

**Affiliations:** 1 Meteorological Research Institute, 1-1 Nagamine, Tsukuba, Ibaraki 305-0052 Japan; 2 Institute for Integrated Radiation and Nuclear Science, Kyoto University (KURNS), 2, Asashiro-Nishi, Kumatori, Sennan, Osaka 590-0494 Japan; 3 Ibaraki University, 2-1-1, Bunkyo, Mito, Ibaraki 310-8512 Japan

**Keywords:** Atmospheric chemistry, Environmental monitoring, Element cycles, Environmental monitoring, Atmospheric chemistry, Geochemistry, Environmental chemistry, Atmospheric chemistry, Biogeochemistry, Biochemistry, Ecology, Microbiology, Plant sciences, Biogeochemistry, Environmental sciences, Environmental social sciences, Planetary science

## Abstract

We have measured artificial radionuclides, such as ^90^Sr and ^137^Cs, in atmospheric depositions since 1957 in Japan. We observed the variations in ^90^Sr and ^137^Cs, which were emitted from atmospheric nuclear tests and nuclear power plant accidents, due to their diffusion, deposition, and resuspension. In March 2011, the Fukushima Daiichi Nuclear Power Plant accident occurred in Japan, and significant increases in ^90^Sr and ^137^Cs were detected at our main site in Tsukuba, Ibaraki. Our continual observations revealed that the ^137^Cs monthly deposition rate in 2018 declined to ~ 1/8100 of the peak level, but it remained more than ~ 400 times higher than that before the accident. Chemical analysis suggested that dust particles were the major carriers of ^90^Sr and ^137^Cs during the resuspension period at our main site. Presently, the effective half-life for ^137^Cs deposition due to radioactive decay and other environmental factors is 4.7 years. The estimation suggests that approximately 42 years from 2011 are required to reduce the atmospheric ^137^Cs deposition to a state similar to that before the accident. The current ^90^Sr deposition, on the other hand, shows the preaccident seasonal variation, and it has returned to the same radioactive level as that before the accident.

## Introduction

**Figure 1:**
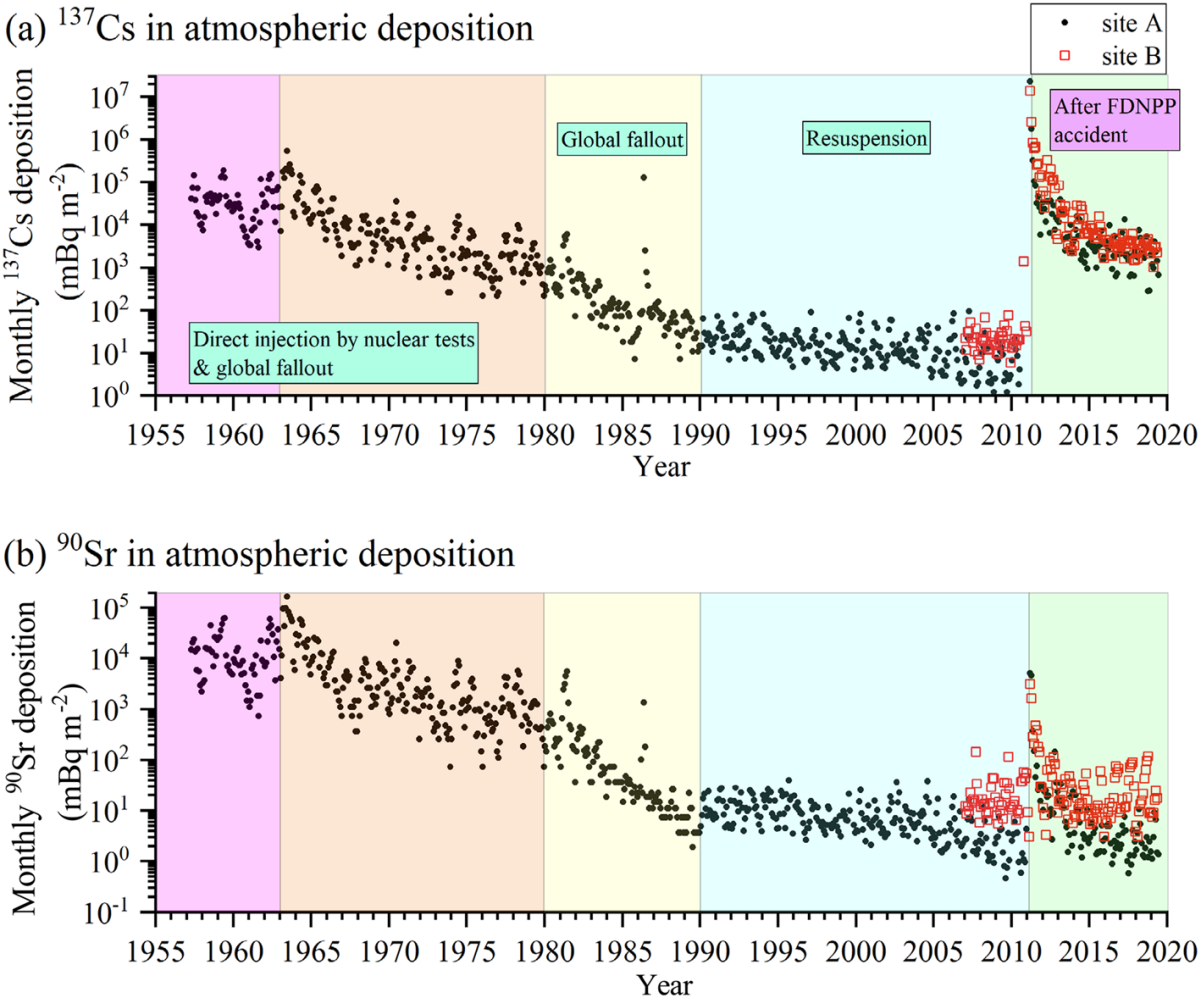
Historical observation of the activity of (**b**) ^90^Sr and (**a**) ^137^Cs in atmospheric depositions (mBq m^−2^) and the change from 1957 to 2019 at site A (closed black circles) and that after 2007 at site B (open red squares).

Atmospheric nuclear tests and nuclear power plant accidents have released artificial radionuclides into the atmosphere, land surface, and ocean. No artificial radionuclides occurred in the environment before 1945, and human activities have led to increases in environmental radioactivity levels. Thus, the monitoring of artificial radionuclides has been a global assignment^1,2^. We have continuously monitored artificial radionuclides in atmospheric depositions for more than 63 years in the Kanto areas around Tokyo, Japan. Our long-term observations clarified the historical variations in artificial radionuclides in atmospheric depositions as a result of nuclear tests and their atmospheric transport and circulation from the 1950s to the 1970s^3–8^. For example, after the Partial Test Ban Treaty (PTBT) in 1963, atmospheric radionuclide deposition from the stratosphere, called global fallout, started to decline. However, the decline of the deposition rate was slowed because China and France continued nuclear tests until 1980. After the last nuclear test in 1980, the decrease rate increased until ~ 1990 (Fig. 1). In 1986, the Chernobyl accident caused a temporary increase in radionuclide deposition^9–11^. From ~ 1990 until March 2011, the decrease of the deposition rate was slowed again because of the change in radionuclide deposition processes, i.e., resuspension of artificial radionuclides hosted by local and remote dust particles^12–15^. These long-term observations of atmospheric deposition have demonstrated that the radionuclide changes in the environment depend on both global and local phenomena. The radionuclides in atmospheric deposition continued to decrease even after the cessation of their direct emissions.

In March 2011, an earthquake with a magnitude of 9.0 occurred and the subsequent tsunami severely damaged the Fukushima Daiichi Nuclear Power Plant (FDNPP). The accident resulted in enormous emissions of artificial radionuclides including ^90^Sr, ^134^Cs, and ^137^Cs (radiocesium) into the atmosphere and ocean^16–21^. Studies have estimated the amount of radioactive materials released from the accident^16,19,22^ and their geographic distributions^23–25^. Other studies showed the chemical and physical properties of the carriers of radionuclides, such as glassy particles^26,27^ and sulfate^28^, and estimated the resuspension processes of ^137^Cs in the atmosphere^29–36^ through dust suspensions^32–34^ or emissions of bioaerosols^34–36^.

The radionuclides released into the environment eventually decline due to radioactive decay and other environmental processes. The rate of radioactive decay is inversely proportional to the respective physical half-life, which is 28.9, 2.1, and 30.2 years for ^90^Sr, ^134^Cs, and ^137^Cs, respectively^37,38^. However, the rate of decline due to environmental removal processes is complex and depends on the weather, environment, and physical and chemical properties of radionuclides. It is crucial to understand the time scale of environmental decay to predict the fate of radioactive materials from accidents and to evaluate their long-term influences on the environment and human health. Hence, this study aims (1) to show our long-term observation results, (2) to estimate the current resuspension carriers of radionuclides, and (3) to evaluate their environmental decay period. To achieve this goal, we measured the radioactivities of ^90^Sr, ^134^Cs, and ^137^Cs and stable elements and isotopes (Na, Mg, Al, K, Ca, Ti, Mn, Fe, Ni, Cu, Zn, Sr, Ba, ^9^Be, ^133^Cs, ^232^Th, and ^238^U) of monthly atmospheric deposition samples collected at two sites in different environments: suburban site A and mountain site B (Supplementary Fig. S1).

## Results and discussion

### Changes in radioactivity in atmospheric depositions after the accident

In March 2011, ^134^Cs was detected with the same activity as that of ^137^Cs. As ^134^Cs had not been detected before the accident except during the emission period resulting from the Chernobyl accident in 1986^11,40,41^, the observed ^134^Cs/^137^Cs ratio verified that the only source of ^134^Cs and ^137^Cs was the FDNPP (Supplementary Fig. S2). Our atmospheric aerosol samples indicated that at least three plumes resulting from the FDNPP accident passed across site A (Supplementary Fig. S3). When these plumes arrived at site A, the activities of ^90^Sr and ^137^Cs in atmospheric deposition increased to 2.7 × 10^3^ and 3.2 × 10^6^ times, respectively, higher than those before the accident (between July 2009 and June 2010) (Fig. 1). The ^137^Cs/^90^Sr activity ratio calculated from our observational results in March 2011 was 4.5 × 10^3^. This large difference in the rate of increase between ^90^Sr and ^137^Cs reflects the discrepancy between their emission rates, i.e., the total amounts of ^90^Sr and ^137^Cs released were estimated as 0.02 PBq^39^ and 14.5 PBq^23^, respectively. These estimations indicated that the ^90^Sr emission level was much lower than that of ^137^Cs. The monthly ^137^Cs deposition peak due to the FDNPP accident (2.31 × 10^4^ Bq m^−2^) was much higher than those resulting from nuclear weapon tests (548 Bq m^−2^; June 1963) and the Chernobyl accident (131 Bq m^−2^; May 1986) (Fig. 1a). On the other hand, the ^90^Sr activity due to the FDNPP accident (5.2 Bq m^−2^) was lower than that due to the nuclear tests in the 1960s (170 Bq m^−2^; June 1963) (Fig. 1b). For comparison, the average ^137^Cs values in atmospheric depositions before the FDNPP accident (between July 2009 and June 2010) were 7.0 (1.2–22.5) mBq m^−2^ at site A and 25.0 (6.1–76.4) mBq m^−2^ at site B, while those for ^90^Sr amounted to 1.9 (ranging from not detectable (N.D.)–6.0) mBq m^−2^ at site A and 26.0 (6.5–116.8) mBq m^−2^ at site B. The possible causes of the higher depositions rates at site B than those at site A are the differences in altitude (site A: 40 m; site B: ~ 1390 m) and local environmental effects (site A: open area; site B: surrounded by forestland).

**Figure 2:**
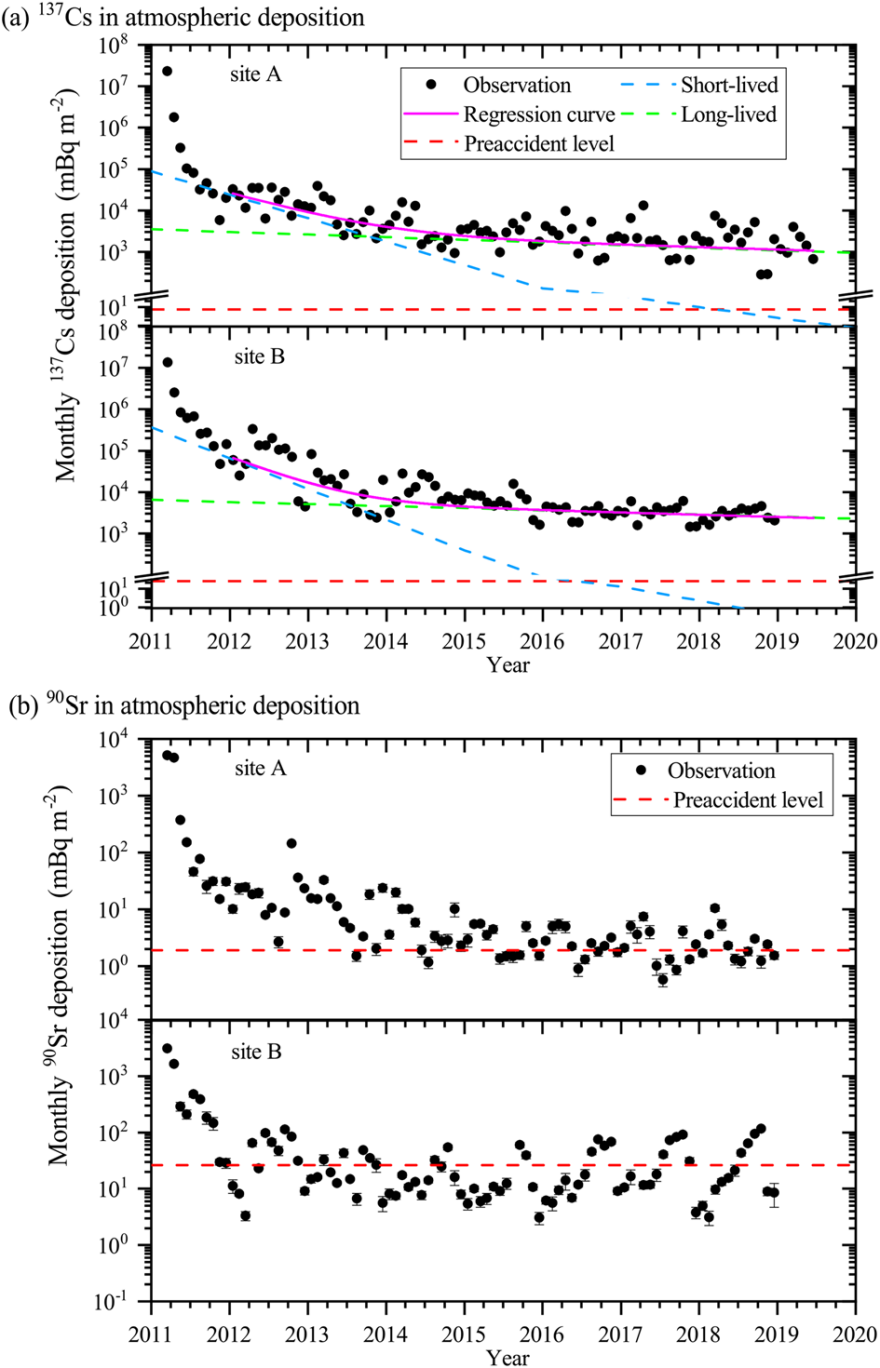
Activity of ^90^Sr and ^137^Cs in atmospheric depositions after the FDNPP accident from 2011 to 2018. (**a**) Cesium-137 in atmospheric depositions. (**b**) Strontium-90 in atmospheric depositions. The black points indicate the observational results. In panel (a), the pink lines indicate the regression curves. The green and blue curves indicate the exponential curves obtained via multiple exponential fitting. The red lines indicate the preaccident levels (the average monthly deposition between June 2009 and July 2010).

The activity of ^90^Sr and ^137^Cs in atmospheric depositions and that of ^137^Cs in aerosol samples rapidly decreased after the first surge in March 2011 (Fig. 2 and Supplementary Fig. S3). The decrease rate of radioactivity in atmospheric depositions at site A was due to the change in radionuclide emission, transport, and deposition processes^29^. We classify the period after the FDNPP accident into three phases. The first phase is dominated by direct emissions (March 2011), the second phase is dominated by tropospheric circulation and removal (from April to December 2011), and the third phase is dominated by resuspension (after January 2012). In the first phase, the direct discharge/emission of radioactive materials during the FDNPP accident and meteorological conditions governed the radionuclide concentration in the environment^29,42–44^. In the second phase, tropospheric transport of the radioactive materials remaining in the atmosphere after the FDNPP accident and their removal processes dominated atmospheric depositions^17,29^. The third phase (after January 2012) mainly depended on the resuspension of radioactive materials^29–31,33–36^. For comparison, the corresponding decrease rates (first, second, and third phases) resulting from the Chernobyl accident were shorter than those resulting from the FDNPP accident (for more discussion details, please refer to Supplementary Fig. S4 and the text). More discussions regarding the first and second phases were also presented in previous studies^29,34^, and hence the scope of the present study is restricted to the third phase.

The latest average monthly ^137^Cs atmospheric depositions in 2018 at sites A and B reached ~ 1/8100 (2.9 Bq m^−2^) and ~ 1/4500 (3.0 Bq m^−2^), respectively, with regard to the peak levels after the accident. But these levels were still ~ 400 and ~ 130 times, respectively, higher than those before the accident (Figs. 1a and 2a, respectively). On the other hand, the ^90^Sr depositions in 2018 amounted to 3.0 (1.2–10.5) mBq m^−2^ and 33.8 (3.1–117) mBq m^−2^ at sites A and B, respectively (Figs. 1b and 2b, respectively). These ^90^Sr deposition levels were almost at the same level as the preaccident deposition levels, and we concluded that the ^90^Sr deposition levels at our observation sites had returned to the preaccident levels in at least 2015 (Fig. 2b).

**Figure 3:**
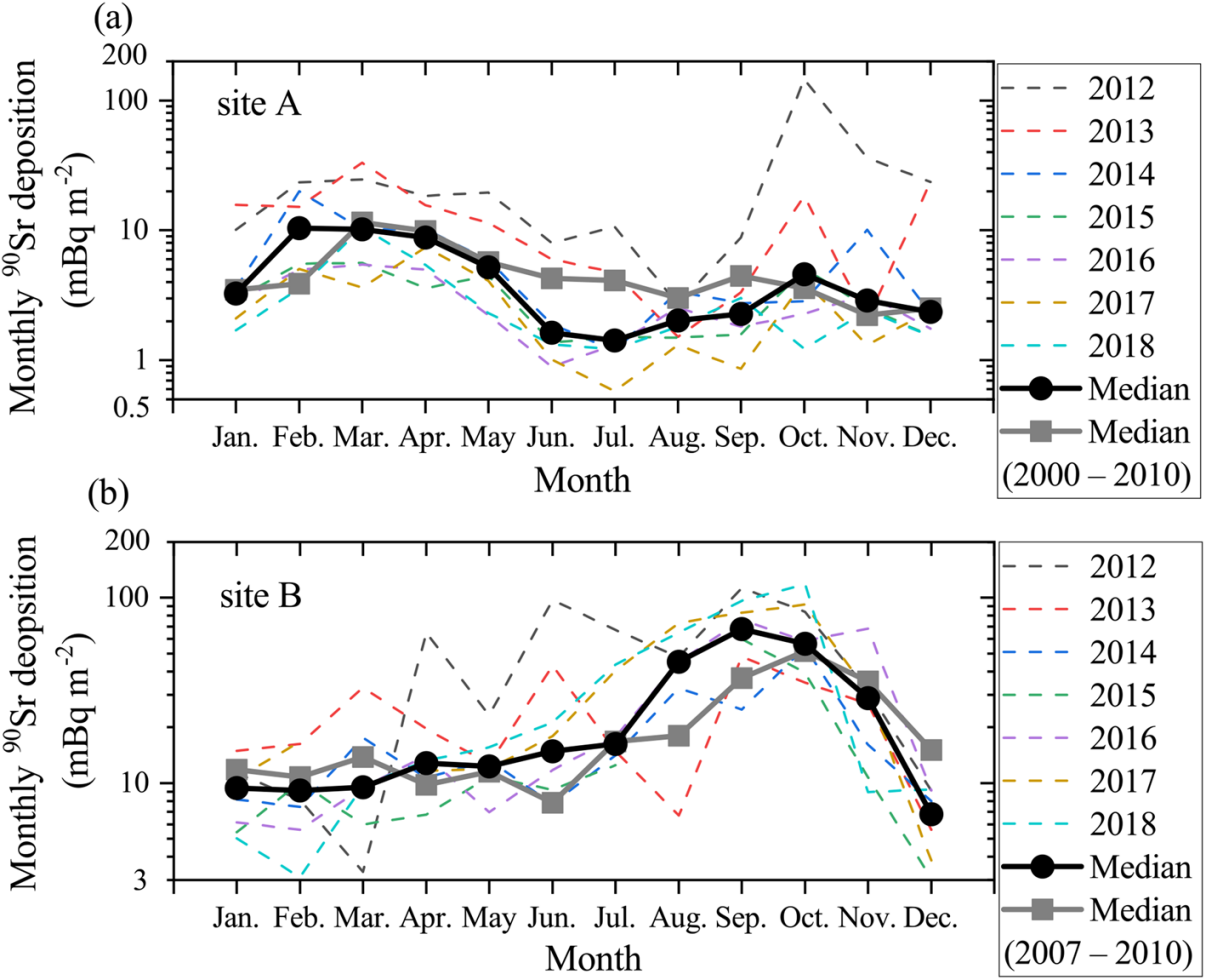
Seasonal changes in ^90^Sr deposition from 2012 to 2018 at (**a**) site A and (**b**) site B. The black curves indicate the median values in each month after the FDNPP accident (from 2012 to 2018). The gray curves indicate those before the accident (from 2000 to 2010 at site A and from 2007 to 2010 at site B).

Before the FDNPP accident, the ^90^Sr and ^137^Cs activity in atmospheric deposition showed seasonal variations (Fig. 3 and Supplementary Figs. S5 and S6). The ^137^Cs deposition value peaks in spring (April) at site A. On the other hand, it peaks twice in May and September at site B (Supplementary Figs. S4 and S5). Similarly, ^90^Sr deposition reaches peak values during the spring season (March and April) at site A and during the fall season (September and October) at site B (Fig. 3). Studies have suggested that the ^90^Sr and ^137^Cs deposition peaks during the spring season at site A are caused by local and long-range transported dust particles^14,15,34,45,46^.

After the FDNPP accident, direct emissions and their tropospheric removal processes governed the ^90^Sr and ^137^Cs activity in atmospheric depositions at sites A and B, and seasonal variations were not apparent in the first and second phases (Fig. 2). After 2012 (in the third phase), although the mean ^137^Cs deposition value at site A had slightly increased in spring (peaking in April), no seasonal variations in ^137^Cs at either site were observed (Fig. 2 and Supplementary Figs. S5 and S6). After 2014, in contrast, the seasonal variations in the ^90^Sr radioactivity in atmospheric deposition at both sites showed similar trends to those before the accident (Figs. 2 and 3).

### Possible carriers of ^90^Sr and ^137^Cs at sites A and B

**Figure 4:**
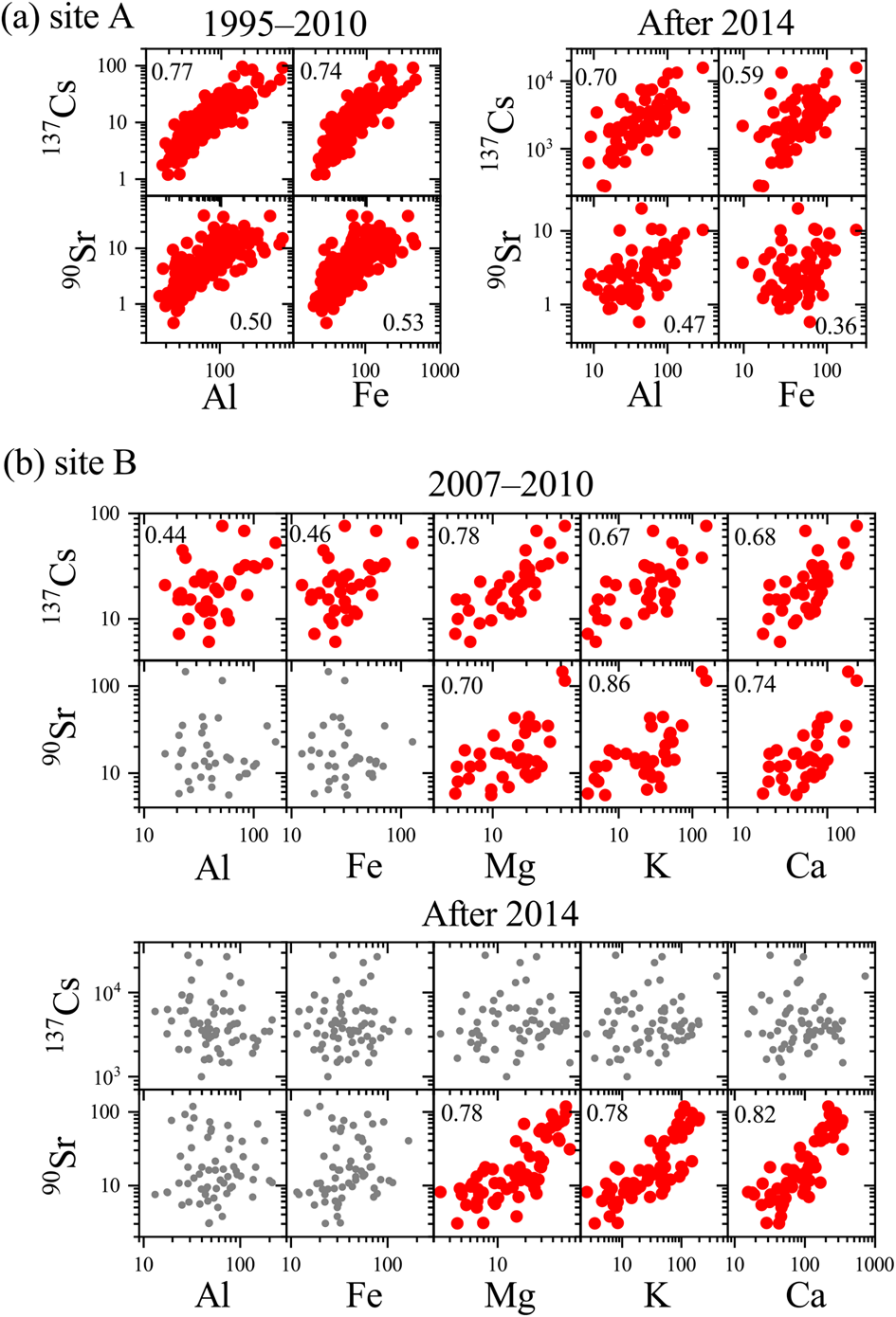
Correlations between radionuclides and stable elements at sites (**a**) A and (**b**) B. The units for ^90^Sr and ^137^Cs are mBq m^−2^, and those for the stable elements are mg m^−2^. The red points reveal that the correlations are significant (*p* < 0.05) based on the correlation coefficient values. The gray points show that the correlations are not significant (*p* ≥ 0.05).

The radionuclides in the atmosphere are generally carried by aerosol particles (host particles) emitted through, for example, geochemical and biological cycles. The correlations between dust components (e.g., Al and Fe) and radionuclides (^90^Sr and ^137^Cs) within the collected samples before the accident suggest that mineral dust particles are the dominant carriers of these radionuclides at site A (Fig. 4a). Previous studies have also demonstrated that the sources of these radionuclides are mainly resuspension of contaminated dust originating from long-range transport (large-scale phenomenon) and neighboring areas (local-scale phenomenon)^14,15,33,34,45,46^. After the accident, chemical analysis results indicate that dust particles are the dominant carriers of ^90^Sr and ^137^Cs at site A, except from 2012 to 2014 for ^90^Sr when the contributions from the accident were high (Fig. 2).

The correlations between the dust components and radionuclides after the accident at site B were poor (Fig. 4b). However, the ^90^Sr activity showed correlations with inorganic salts such as Mg, K, and Ca at site B. Scanning electron microscopy (SEM) observation exhibited the presence of inorganic salt particles including KCl, NaCl, and CaSO_4_ in dried deposition samples (Supplementary Fig. S7). Although these salt particles had possibly crystallized during the preparation of the atmospheric deposition samples, it is probable that ^90^Sr coexists with these components in the environment as they are abundantly present in the samples. Studies have indicated that the biological cycle may be a source of these inorganic elements in forests^47–49^, i.e., the Mg, K, and Ca concentrations in throughfall depositions increase in forests due to foliar leaching. As Sr exhibits a similar geochemical behavior to that of Ca, the occurrence of Sr could be synchronous to that of Ca in the neighboring forest.

Before the accident, the ^137^Cs activity at site B showed positive correlations with major mineral dust components such as Mg, Mn, Ca, K, Fe, and Al (Fig. 4), suggesting that dust particles were the dominant host particles for ^137^Cs. However, no significant correlation was detected between mineral dust and the ^137^Cs activity after 2014. Previous studies have suggested that bioaerosols contribute to the resuspension of ^137^Cs at forest sites in the contaminated area within the evacuation zone in Fukushima Prefecture^35,36^. Thus, it is possible that bioaerosols carry ^137^Cs at site B. The differences between the possible carriers may cause the observed differences in the activity ratios of sites B and A (R_B/A_) for ^90^Sr and ^137^Cs deposition after the accident (Supplementary Fig. S7).

### Estimation of the environmental decrease in ^137^Cs

With the use of regression curve fitting of the activity of ^137^Cs in atmospheric deposition, we estimated its effective half-life due to radioactive decay and environmental removal processes (Fig. 2). We adopted a single-exponential function before the accident from January 1990 to July 2010 and a multiple exponential function after the accident (2012–2018; the resuspension phase). The detailed method of the calculation is described in the Supplementary Information.

The effective half-lives of the short- and long-lived components (t_1_ and t_2_, respectively) of the ^137^Cs deposition were 195 days and 4.7 years, respectively, at site A, and those at site B were 148 days and 5.9 years, respectively. Interchange of the predominant short- and long-lived components occurred during the period between September and December 2013 (Fig. 2). Our estimation of the effective half-life of the long-lived component at site A is longer than the estimation by the previous study (~ 1.1 years)^29^ possibly because our estimation 1) excluded the direct emission period and 2) extended observation data by the end of 2018. The effective half-life of the long-lived component of ^137^Cs at site A after the FDNPP accident is shorter than that before the accident (8.5 years). There are two possible reasons for the difference between the effective half-lives before and after the accident. First, the dominant resuspension processes are different before and after the accident. Second, the elapsed time after contaminations is different between the pre and postaccident periods, i.e., more than 30 years had passed for the analysis period before the accident since the last atmospheric nuclear test, on the other hand, only 8 years had passed since the significant pollution after the FDNPP accident.

The above estimated effective half-lives imply that, based on the atmospheric ^137^Cs deposition level, ~ 42 and ~ 48 years will be required from the year of the accident to reach the preaccident level at sites A and B, respectively. These estimates contain uncertainties due to the short observation period compared to the effective half-life before the FDNPP accident (8.5 years). A better understanding of the carriers, resuspension processes, and environmental circulation conditions of radionuclides is needed to confirm the above estimates. The radionuclide decreasing trend may change in the future if resuspension process, biological recycling, and their carriers changed. Finally, our observations only pertain to atmospheric deposition and provide limited features of the environmental radionuclide cycle. The contamination in other fields, such as surface soils, forests, and oceans, will exhibit different effective half-lives. Nevertheless, our continuous observations of the radionuclides in atmospheric deposition before and after the accident enable the evaluation of the atmospheric phases and the changes in various processes to regain the environmental conditions before the nuclear power plant accident.

## Material and methods

We collected monthly atmospheric deposition samples at two sites: a suburban site in the Kanto Plain (site A; 36.1°N, 140.1° E) and a mountain site in the northwestern corner of the Kanto Plain (site B, 36.5°N, 138.9° E) (Supplementary Fig. S1). Site A is the main observation base and was established in 1980 at the Meteorological Research Institute (MRI), Tsukuba, Japan. From 1957 to 1980, the main observation site was located in Koenji, Tokyo, which was shifted to the current base due to the move of the MRI in 1980^50^. Sampling trays were placed on the rooftop of one-story (1980–2011) and six-story buildings (2011–) on the MRI campus. Site B was established in 2007 at the top of the Mt. Haruna (1390 m above sea level), Gunma, Japan. Sites A and B are 170 and 250 km away, respectively, from the FDNPP.

Atmospheric deposition samples, which include both rain (wet deposition) and dry deposition, were collected using the above plastic trays with a total open area of 1–4 m^2^, depending on the sampling period. The samples were sieved through a 106 µm mesh. The deposition samples were dried using rotary evaporators (Eyela NE-12, Tokyo Rikakikai Ltd., Japan) and evaporating dishes followed by weight measurements. After March 2011, we collected aerosol samples using high-volume air samplers (HV-1000F, Shibata Scientific Technology Ltd., Japan) on quartz fiber filters (QR100, Advantech Ltd., USA) at a flow rate of 700 L per minute to observe the atmospheric radioactivity concentration.

The activity of radiocesium was measured by Ge semiconductor detectors (of the coaxial type from ORTEC EG&G and Eurisys) coupled with a computed spectrometric analyzer (Oxford-Tennelec Multiport or Seiko EG&G 92x) using a maximum live time of 10^6^ s after the FDNPP accident. After the radiocesium measurement, ^90^Sr was radiochemically separated, purified, and solidified as Sr-carbonate precipitates. After leaving the sample for several weeks in order to achieve ^90^Sr and ^90^Y radioequilibrium, the β-activity was measured with an alpha/beta counting system (Tennelec LB5100, Mirion Technologies, USA) using a maximum live time of 10^3^ min. The detection limits were 1.55 and 39.6 mBq m^−2^ for ^90^Sr and ^137^Cs, respectively, in the deposition samples, which were, obtained by multiplying each counting error measured in 2018 by three. Details on the sample preparation and measurement methods have been described in a previous study^51^.

The stable elements (Na, Mg, Al, K, Ca, Ti, Mn, Fe, Ni, Cu, Zn, Sr, and Ba) and isotopes (^9^Be, ^133^Cs, ^232^Th, and ^238^U) were measured by inductively coupled plasma atomic emission spectrometry (CIROS-120, Rigaku Corp., Japan, or Vista-PRO, Varian Inc., USA) and inductively coupled plasma mass spectrometry (Agilent7500c or Agilent8000, Agilent, Ltd., USA), respectively, based on aliquots of the samples (3.6% in mass) during the acid decomposition processes. The detection limit and quantification values were estimated as three and ten times the standard deviation of ten measurements of 10 ppb standards. An SEM (SU-3500, Hitachi High Technologies Co., Japan) with an energy-dispersive X-ray spectrometer (EDX; E-max 50 mm, Horiba Ltd., Japan) was adopted for chemical and physical analysis of the dried deposition samples.

## Supplementary information

Supplementary Information.
