# Supplementary material for: Temporal variations of 90Sr and 137Cs in atmospheric depositions after the Fukushima Daiichi Nuclear Power Plant accident with long-term observations
Source: Sci Rep. 2020 Dec 10;10:21627. doi: 10.1038/s41598-020-78312-3 (PMC7728751; doi:10.1038/s41598-020-78312-3)
Supplement: Supplementary file 1 — Supplementary Information. [file 41598_2020_78312_MOESM1_ESM.docx]

Temporal variations of ^90^Sr and ^137^Cs in atmospheric depositions after the Fukushima Daiichi Nuclear Power Plant accident with long-term observations

Takeshi Kinase^1,*^, Kouji Adachi^1^, Tsuyoshi Thomas Sekiyama^1^, Mizuo Kajino^1^, Yuji Zaizen^1^, and Yasuhito Igarashi^2,3^

^1^Meteorological Research Institute, 1-1 Nagamine, Tsukuba, Ibaraki, 305-0052, Japan.

^2^Institute for Integrated Radiation and Nuclear Science, Kyoto University (KURNS), 2, Asashiro-Nishi, Kumatori, Sennan, Osaka, 590-0494, Japan

^3^Ibaraki University, 2-1-1, Bunkyo, Mito, Ibaraki, 310-8512, Japan

^*^corresponding author: [tkinase@mri-jma.go.jp](mailto:tkinase@mri-jma.go.jp)

Keywords: Radionuclide, Fallout, Aerosol, Fukushima, Resuspension, Deposition, Strontium, Cesium

**ABSTRACT**

We have measured artificial radionuclides, such as ^90^Sr and ^137^Cs, in atmospheric depositions since 1957 in Japan. We observed the variations in ^90^Sr and ^137^Cs, which were emitted from atmospheric nuclear tests and nuclear power plant accidents, due to their diffusion, deposition, and resuspension. In March 2011, the Fukushima Daiichi Nuclear Power Plant (FDNPP) accident occurred in Japan, and significant increases in ^90^Sr and ^137^Cs were detected at our main site in Tsukuba, Ibaraki. Our continual observations revealed that the ^137^Cs monthly deposition rate in 2018 declined to ~1/8100 of the peak level, but it remained more than ~400 times higher than that before the accident. Chemical analysis suggested that dust particles were the major carriers of ^90^Sr and ^137^Cs during the resuspension period at our main site. Presently, the effective half-life for ^137^Cs deposition due to radioactive decay and other environmental factors is 4.7 years. The estimation suggests that approximately 42 years from 2011 are required to reduce the atmospheric ^137^Cs deposition to a state similar to that before the accident. The current ^90^Sr deposition, on the other hand, shows the preaccident seasonal variation, and it has returned to the same radioactive level as that before the accident.

**Introduction**

Atmospheric nuclear tests and nuclear power plant accidents have released artificial radionuclides into the atmosphere, land surface, and ocean. No artificial radionuclides occurred in the environment before 1945, and human activities have led to increases in environmental radioactivity levels. Thus, the monitoring of artificial radionuclides has been a global assignment^1,2^. We have continuously monitored artificial radionuclides in atmospheric depositions for more than 63 years in the Kanto areas around Tokyo, Japan. Our long-term observations clarified the historical variations in artificial radionuclides in atmospheric depositions as a result of nuclear tests and their atmospheric transport and circulation from the 1950s to the 1970s^3–8^. For example, after the Partial Test Ban Treaty (PTBT) in 1963, atmospheric radionuclide deposition from the stratosphere, called global fallout, started to decline. However, the decline of the deposition rate was slowed because China and France continued nuclear tests until 1980. After the last nuclear test in 1980, the decrease rate increased until ~1990 (Fig. 1). In 1986, the Chernobyl accident caused a temporary increase in radionuclide deposition^9–11^. From ~1990 until March 2011, the decrease of the deposition rate was slowed again because of the change in radionuclide deposition processes, i.e., resuspension of artificial radionuclides hosted by local and remote dust particles^12–15^. These long-term observations of atmospheric deposition have demonstrated that the radionuclide changes in the environment depend on both global and local phenomena. The radionuclides in atmospheric deposition continued to decrease even after the cessation of their direct emissions.

In March 2011, an earthquake with a magnitude of 9.0 occurred and the subsequent tsunami severely damaged the Fukushima Daiichi Nuclear Power Plant (FDNPP). The accident resulted in enormous emissions of artificial radionuclides including ^90^Sr, ^134^Cs, and ^137^Cs (radiocesium) into the atmosphere and ocean^16–21^. Studies have estimated the amount of radioactive materials released from the accident^16,19,22^ and their geographic distributions^23–25^. Other studies showed the chemical and physical properties of the carriers of radionuclides, such as glassy particles^26,27^ and sulfate^28^, and estimated the resuspension processes of ^137^Cs in the atmosphere^29–36^ through dust suspensions^32–34^ or emissions of bioaerosols^34–36^.

The radionuclides released into the environment eventually decline due to radioactive decay and other environmental processes. The rate of radioactive decay is inversely proportional to the respective physical half-life, which is 28.9, 2.1, and 30.2 years for ^90^Sr, ^134^Cs, and ^137^Cs, respectively^37,38^. However, the rate of decline due to environmental removal processes is complex and depends on the weather, environment, and physical and chemical properties of radionuclides. It is crucial to understand the time scale of environmental decay to predict the fate of radioactive materials from accidents and to evaluate their long-term influences on the environment and human health. Hence, this study aims 1) to show our long-term observation results, 2) to estimate the current resuspension carriers of radionuclides, and 3) to evaluate their environmental decay period. To achieve this goal, we measured the radioactivities of ^90^Sr, ^134^Cs, and ^137^Cs and stable elements and isotopes (Na, Mg, Al, K, Ca, Ti, Mn, Fe, Ni, Cu, Zn, Sr, Ba, ^9^Be, ^133^Cs, ^232^Th, and ^238^U) of monthly atmospheric deposition samples collected at two sites in different environments: suburban site A and mountain site B (Supplementary Fig. S1).

**Results and discussion**

**Changes in radioactivity in atmospheric depositions after the accident**

In March 2011, ^134^Cs was detected with the same activity as that of ^137^Cs. As ^134^Cs had not been detected before the accident except during the emission period resulting from the Chernobyl accident in 1986^11,40,41^, the observed ^134^Cs/^137^Cs ratio verified that the only source of ^134^Cs and ^137^Cs was the FDNPP (Supplementary Fig. S2). Our atmospheric aerosol samples indicated that at least three plumes resulting from the FDNPP accident passed across site A (Supplementary Fig. S3). When these plumes arrived at site A, the activities of ^90^Sr and ^137^Cs in atmospheric deposition increased to 2.7×10^3^ and 3.2×10^6^ times, respectively, higher than those before the accident (between July 2009 and June 2010) (Fig. 1). The ^137^Cs/^90^Sr activity ratio calculated from our observational results in March 2011 was 4.5×10^3^. This large difference in the rate of increase between ^90^Sr and ^137^Cs reflects the discrepancy between their emission rates, i.e., the total amounts of ^90^Sr and ^137^Cs released were estimated as 0.02 PBq^39^ and 14.5 PBq^23^, respectively. These estimations indicated that the ^90^Sr emission level was much lower than that of ^137^Cs. The monthly ^137^Cs deposition peak due to the FDNPP accident (2.31 × 10^4^ Bq m^-2^) was much higher than those resulting from nuclear weapon tests (548 Bq m^-2^; June 1963) and the Chernobyl accident (131 Bq m^-2^; May 1986) (Fig. 1a). On the other hand, the ^90^Sr activity due to the FDNPP accident (5.2 Bq m^-2^) was lower than that due to the nuclear tests in the 1960s (170 Bq m^-2^; June 1963) (Fig. 1b). For comparison, the average ^137^Cs values in atmospheric depositions before the FDNPP accident (between July 2009 and June 2010) were 7.0 (1.2–22.5) mBq m^-2^ at site A and 25.0 (6.1–76.4) mBq m^-2^ at site B, while those for ^90^Sr amounted to 1.9 (ranging from not detectable (N.D.)–6.0) mBq m^-2^ at site A and 26.0 (6.5–116.8) mBq m^-2^ at site B. The possible causes of the higher depositions rates at site B than those at site A are the differences in altitude (site A: 40 m; site B: ~1390 m) and local environmental effects (site A: open area; site B: surrounded by forestland).





Figure 1. Historical observation of the activity of (b) ^90^Sr and (a) ^137^Cs in atmospheric depositions (mBq m^-2^) and the change from 1957 to 2019 at site A (closed black circles) and that after 2007 at site B (open red squares).

The activity of ^90^Sr and ^137^Cs in atmospheric depositions and that of ^137^Cs in aerosol samples rapidly decreased after the first surge in March 2011 (Fig. 2 and Supplementary Fig. S3). The decrease rate of radioactivity in atmospheric depositions at site A was due to the change in radionuclide emission, transport, and deposition processes^29^. We classify the period after the FDNPP accident into three phases. The first phase is dominated by direct emissions (March 2011), the second phase is dominated by tropospheric circulation and removal (from April to December 2011), and the third phase is dominated by resuspension (after January 2012). In the first phase, the direct discharge/emission of radioactive materials during the FDNPP accident and meteorological conditions governed the radionuclide concentration in the environment^29,42–44^. In the second phase, tropospheric transport of the radioactive materials remaining in the atmosphere after the FDNPP accident and their removal processes dominated atmospheric depositions^17,29^. The third phase (after January 2012) mainly depended on the resuspension of radioactive materials^29–31,33–36^. For comparison, the corresponding decrease rates (first, second, and third phases) resulting from the Chernobyl accident were shorter than those resulting from the FDNPP accident (for more discussion details, please refer to Supplementary Fig. S4 and the text). More discussions regarding the first and second phases were also presented in previous studies^29,34^, and hence the scope of the present study is restricted to the third phase.

The latest average monthly ^137^Cs atmospheric depositions in 2018 at sites A and B reached ~1/8100 (2.9 Bq m^-2^) and ~1/4500 (3.0 Bq m^-2^), respectively, with regard to the peak levels after the accident. But these levels were still ~400 and ~130 times, respectively, higher than those before the accident (Figs. 1a and 2a, respectively). On the other hand, the ^90^Sr depositions in 2018 amounted to 3.0 (1.2–10.5) mBq m^-2^ and 33.8 (3.1–117) mBq m^-2^ at sites A and B, respectively (Figs. 1b and 2b, respectively). These ^90^Sr deposition levels were almost at the same level as the preaccident deposition levels, and we concluded that the ^90^Sr deposition levels at our observation sites had returned to the preaccident levels in at least 2015 (Fig. 2b).





Figure 2. Activity of ^90^Sr and ^137^Cs in atmospheric depositions after the FDNPP accident from 2011 to 2018. (a) Cesium-137 in atmospheric depositions. (b) Strontium-90 in atmospheric depositions. The black points indicate the observational results. In panel (a), the pink lines indicate the regression curves. The green and blue curves indicate the exponential curves obtained via multiple exponential fitting. The red lines indicate the preaccident levels (the average monthly deposition between June 2009 and July 2010).

Before the FDNPP accident, the ^90^Sr and ^137^Cs activity in atmospheric deposition showed seasonal variations (Fig. 3 and Supplementary Figs. S5 and S6). The ^137^Cs deposition value peaks in spring (April) at site A. On the other hand, it peaks twice in May and September at site B (Supplementary Figs. S4 and S5). Similarly, ^90^Sr deposition reaches peak values during the spring season (March and April) at site A and during the fall season (September and October) at site B (Fig. 3). Studies have suggested that the ^90^Sr and ^137^Cs deposition peaks during the spring season at site A are caused by local and long-range transported dust particles^14,15,34,45,46^.

After the FDNPP accident, direct emissions and their tropospheric removal processes governed the ^90^Sr and ^137^Cs activity in atmospheric depositions at sites A and B, and seasonal variations were not apparent in the first and second phases (Fig. 2). After 2012 (in the third phase), although the mean ^137^Cs deposition value at site A had slightly increased in spring (peaking in April), no seasonal variations in ^137^Cs at either site were observed (Fig. 2 and Supplementary Figs. S5 and S6). After 2014, in contrast, the seasonal variations in the ^90^Sr radioactivity in atmospheric deposition at both sites showed similar trends to those before the accident (Figs. 2 and 3).





Figure 3. Seasonal changes in ^90^Sr deposition from 2012 to 2018 at (a) site A and (b) site B. The black curves indicate the median values in each month after the FDNPP accident (from 2012 to 2018). The gray curves indicate those before the accident (from 2000 to 2010 at site A and from 2007 to 2010 at site B).

**Possible carriers of ^90^Sr and ^137^Cs at sites A and B**

The radionuclides in the atmosphere are generally carried by aerosol particles (host particles) emitted through, for example, geochemical and biological cycles. The correlations between dust components (e.g., Al and Fe) and radionuclides (^90^Sr and ^137^Cs) within the collected samples before the accident suggest that mineral dust particles are the dominant carriers of these radionuclides at site A (Fig. 4a). Previous studies have also demonstrated that the sources of these radionuclides are mainly resuspension of contaminated dust originating from long-range transport (large-scale phenomenon) and neighboring areas (local-scale phenomenon)^14,15,33,34,45,46^. After the accident, chemical analysis results indicate that dust particles are the dominant carriers of ^90^Sr and ^137^Cs at site A, except from 2012–2014 for ^90^Sr when the contributions from the accident were high (Fig. 2).





Figure 4. Correlations between radionuclides and stable elements at sites (a) A and (b) B. The units for ^90^Sr and ^137^Cs are mBq m^-2^, and those for the stable elements are mg m^-2^. The red points reveal that the correlations are significant (*p*<0.05) based on the correlation coefficient values. The gray points show that the correlations are not significant (*p*≥0.05).

The correlations between the dust components and radionuclides after the accident at site B were poor (Fig. 4b). However, the ^90^Sr activity showed correlations with inorganic salts such as Mg, K, and Ca at site B. Scanning electron microscopy (SEM) observation exhibited the presence of inorganic salt particles including KCl, NaCl, and CaSO_4_ in dried deposition samples (Supplementary Fig. S7). Although these salt particles had possibly crystallized during the preparation of the atmospheric deposition samples, it is probable that ^90^Sr coexists with these components in the environment as they are abundantly present in the samples. Studies have indicated that the biological cycle may be a source of these inorganic elements in forests^47–49^, i.e., the Mg, K, and Ca concentrations in throughfall depositions increase in forests due to foliar leaching. As Sr exhibits a similar geochemical behavior to that of Ca, the occurrence of Sr could be synchronous to that of Ca in the neighboring forest.

Before the accident, the ^137^Cs activity at site B showed positive correlations with major mineral dust components such as Mg, Mn, Ca, K, Fe, and Al (Fig.4), suggesting that dust particles were the dominant host particles for ^137^Cs. However, no significant correlation was detected between mineral dust and the ^137^Cs activity after 2014. Previous studies have suggested that bioaerosols contribute to the resuspension of ^137^Cs at forest sites in the contaminated area within the evacuation zone in Fukushima Prefecture^35,36^. Thus, it is possible that bioaerosols carry ^137^Cs at site B. The differences between the possible carriers may cause the observed differences in the activity ratios of sites B and A (R_B/A_) for ^90^Sr and ^137^Cs deposition after the accident (Supplementary Fig. S7).

**Estimation of the environmental decrease in ^137^Cs**

With the use of regression curve fitting of the activity of ^137^Cs in atmospheric deposition, we estimated its effective half-life due to radioactive decay and environmental removal processes (Fig. 2). We adopted a single-exponential function before the accident from January 1990 to July 2010 and a multiple exponential function after the accident (2012–2018; the resuspension phase). The detailed method of the calculation is described in the Supplementary Information.

The effective half-lives of the short- and long-lived components (t_1_ and t_2_, respectively) of the ^137^Cs deposition were 195 days and 4.7 years, respectively, at site A, and those at site B were 148 days and 5.9 years, respectively. Interchange of the predominant short- and long-lived components occurred during the period between September and December 2013 (Fig. 2). Our estimation of the effective half-life of the long-lived component at site A is longer than the estimation by the previous study (~1.1 years)^29^ possibly because our estimation 1) excluded the direct emission period and 2) extended observation data by the end of 2018. The effective half-life of the long-lived component of ^137^Cs at site A after the FDNPP accident is shorter than that before the accident (8.5 years). There are two possible reasons for the difference between the effective half-lives before and after the accident. First, the dominant resuspension processes are different before and after the accident. Second, the elapsed time after contaminations is different between the pre and postaccident periods, i.e., more than 30 years had passed for the analysis period before the accident since the last atmospheric nuclear test, on the other hand, only 8 years had passed since the significant pollution after the FDNPP accident.

The above estimated effective half-lives imply that, based on the atmospheric ^137^Cs deposition level, ~42 and ~48 years will be required from the year of the accident to reach the preaccident level at sites A and B, respectively. These estimates contain uncertainties due to the short observation period compared to the effective half-life before the FDNPP accident (8.5 years). A better understanding of the carriers, resuspension processes, and environmental circulation conditions of radionuclides is needed to confirm the above estimates. The radionuclide decreasing trend may change in the future if resuspension process, biological recycling, and their carriers changed. Finally, our observations only pertain to atmospheric deposition and provide limited features of the environmental radionuclide cycle. The contamination in other fields, such as surface soils, forests, and oceans, will exhibit different effective half-lives. Nevertheless, our continuous observations of the radionuclides in atmospheric deposition before and after the accident enable the evaluation of the atmospheric phases and the changes in various processes to regain the environmental conditions before the nuclear power plant accident.

**Material and methods**

We collected monthly atmospheric deposition samples at two sites: a suburban site in the Kanto Plain (site A; 36.1°N, 140.1° E) and a mountain site in the northwestern corner of the Kanto Plain (site B, 36.5°N, 138.9° E) (Supplementary Fig. S1). Site A is the main observation base and was established in 1980 at the Meteorological Research Institute (MRI), Tsukuba, Japan. From 1957 to 1980, the main observation site was located in Koenji, Tokyo, which was shifted to the current base due to the move of the MRI in 1980^50^. Sampling trays were placed on the rooftop of one-story (1980–2011) and six-story buildings (2011–) on the MRI campus. Site B was established in 2007 at the top of the Mt. Haruna (1390 m above sea level), Gunma, Japan. Sites A and B are 170 and 250 km away, respectively, from the FDNPP.

Atmospheric deposition samples, which include both rain (wet deposition) and dry deposition, were collected using the above plastic trays with a total open area of 1–4 m^2^, depending on the sampling period. The samples were sieved through a 106 µm mesh. The deposition samples were dried using rotary evaporators (Eyela NE-12, Tokyo Rikakikai Ltd., Japan) and evaporating dishes followed by weight measurements. After March 2011, we collected aerosol samples using high-volume air samplers (HV-1000F, Shibata Scientific Technology Ltd., Japan) on quartz fiber filters (QR100, Advantech Ltd., USA) at a flow rate of 700 liters per minute to observe the atmospheric radioactivity concentration.

The activity of radiocesium was measured by Ge semiconductor detectors (of the coaxial type from ORTEC EG&G and Eurisys) coupled with a computed spectrometric analyzer (Oxford-Tennelec Multiport or Seiko EG&G 92x) using a maximum live time of 10^6^ seconds after the FDNPP accident. After the radiocesium measurement, ^90^Sr was radiochemically separated, purified, and solidified as Sr-carbonate precipitates. After leaving the sample for several weeks in order to achieve ^90^Sr and ^90^Y radioequilibrium, the β-activity was measured with an alpha/beta counting system (Tennelec LB5100, Mirion Technologies, USA) using a maximum live time of 10^3^ minutes. The detection limits were 1.55 and 39.6 mBq m^-2^ for ^90^Sr and ^137^Cs, respectively, in the deposition samples, which were, obtained by multiplying each counting error measured in 2018 by three. Details on the sample preparation and measurement methods have been described in a previous study^51^.

The stable elements (Na, Mg, Al, K, Ca, Ti, Mn, Fe, Ni, Cu, Zn, Sr, and Ba) and isotopes (^9^Be, ^133^Cs, ^232^Th, and ^238^U) were measured by inductively coupled plasma atomic emission spectrometry (CIROS-120, Rigaku Corp., Japan, or Vista-PRO, Varian Inc., USA) and inductively coupled plasma mass spectrometry (Agilent7500c or Agilent8000, Agilent, Ltd., USA), respectively, based on aliquots of the samples (3.6% in mass) during the acid decomposition processes. The detection limit and quantification values were estimated as three and ten times the standard deviation of ten measurements of 10 ppb standards. An SEM (SU-3500, Hitachi High Technologies Co., Japan) with an energy-dispersive X-ray spectrometer (EDX; E-max 50 mm, Horiba Ltd., Japan) was adopted for chemical and physical analysis of the dried deposition samples.

**REFERENCES**

1. United Nations Scientific Committee on the Effects of Atomic Radiation (UNSCEAR). UNSCEAR 2000 Report to the General Assembly, with Scientific Annexes. *UNSCEAR* https://www.unscear.org/docs/publications/2000/UNSCEAR_2000_Report_Vol.I.pdf (2000).
2. United Nations Scientific Committee on the Effects of Atomic Radiation (UNSCEAR). UNSCEAR 2013 Report to the General Assembly, with Scientific Annexes, Volume2 Scientific Annex B. *UNSCEAR* https://www.unscear.org/docs/publications/2013/UNSCEAR_2013_Report_Vol.II.pdf (2013)
3. Hirose, K., Sugimura, Y. & Katsuragi, Y. ^90^Sr and ^239+240^Pu in the surface air in Japan: their concentrations and size distributions. *Pap. Meteorol. Geophys.* **37** (4), 255－269, DOI: https://doi.org/10.2467/mripapers.37.255 (1986).
4. Katsuragi, Y. A study of ^90^Sr fallout in Japan, *Pap. Meteorol. Geophys.* **33** (4), 277－291, DOI: https://doi.org/10.2467/mripapers.33.277 (1983).
5. Katsuragi, Y. & Michio, A. Seasonal variation of Sr-90 fallout in Japan through the end of 1983, *Pap. Meteorol. Geophys.* **37** (1), 15－36, DOI: https://doi.org/10.2467/mripapers.37.15 (1986).
6. Miyake, Y. The artificial radioactivity in rain water observed in Japan, from Autumn 1954 to Spring 1955. *Pap. Meteorol. Geophys.* **5** (2), 53－62, DOI: https://doi.org/10.2467/mripapers1950.6.1_26 (1955).
7. Miyake, Y., Saruhashi, K., Katsuragi, Y., Kanazawa, T. & Tsunogai, S. Deposition of Sr-90 and Cs-137 in Tokyo through the end of July 1963. *Pap. Meteorol. Geophys.* **14**, 58－65, DOI: https://pdfs.semanticscholar.org/8211/5a14c17733a024fb12fc7bd070b12e836319.pdf (1963).
8. Miyake, Y., Katsuragi, Y. & Sugimura, Y. A study on Plutonium fallout. *J. Geophys. Res.* **75** (12), 2329–2330, https://agupubs.onlinelibrary.wiley.com/doi/epdf/10.1029/JC075i012p02329 (1970).
9. Aoyama, M., Hirose, K., Suzuki, Y., Inoue, H. & Sugimura, Y. High level radioactive nuclides in Japan in May. *Nature* **321**, 819–20, DOI: https://doi.org/10.1038/321819a0 (1986).
10. Aoyama, M., Hirose, K. & Sugimura, Y. Deposition of gamma-emitting nuclides in Japan after the reactor-IV accident at Chernobyl. *J. Radioanal. Nucl. Chem.,* **116**, 291–306. DOI: https://doi.org/10.1007/BF02035773 (1987).
11. Hirose, K. Takatani, S. & Michio, A. Wet deposition of radionuclides derived from the Chernobyl accident. *J. Atmos. Chem.* **17**, 61–71, DOI: https://doi.org/10.1007/BF00699114 (1993).
12. Igarashi, Y., Otsuji-Hatori, M. & Hirose, K. Recent deposition of ^90^Sr and ^137^Cs observed in Tsukuba. *J. Environ. Radioact.* **31** (2), 157–169, DOI: https://doi.org/10.1016/0265-931X(96)88491-8 (1996).
13. Igarashi, Y., Aoyama, M., Miyao, T., Hirose, K. & Tomita, M. Anomalous ^90^Sr deposition during the fall, 1995 at MRI, Tsukuba, Japan. *J. Radioanal. Nucl. Chem.* **239** (3), 539–542, DOI: https://doi.org/10.1007/bf02349065 (1999).
14. Igarashi, Y et al. Resuspension: decadal monitoring time series of the anthropogenic radioactivity deposition in Japan. *J. Radiat. Res.* **44**, 319–328, DOI: https://doi.org/10.1269/jrr.44.319 (2003).
15. Igarashi, Y. et al. Possible change in Asian dust source suggested by atmospheric anthropogenic radionuclides during the 2000s. *Atmos. Environ.* **43**, 2971–2980, DOI: https://doi.org/10.1016/j.atmosenv.2009.02.018 (2009).
16. Chino, M. et al. Preliminary Estimation of Release Amounts of ^131^I and ^137^Cs Accidentally Discharged from the Fukushima Daiichi Nuclear Power Plant into the Atmosphere, *J. Nucl. Sci. Technol.* **48** (7), 1129–1134, DOI: https://doi.org/10.1080/18811248.2011.9711799 (2011).
17. Hirose, K. Fukushima Daiichi Nuclear Plant accident: Atmospheric and oceanic impacts over the five years, *J. Environ. Radioact.* **157**, 113–130, DOI: https://doi.org/10.1016/j.jenvrad.2016.01.011 (2016).
18. Masson, O. et al. Variation in airborne ^134^Cs, ^137^Cs, particulate ^131^I and ^7^Be maximum activities at high-altitude European locations after the arrival of Fukushima-labeled air masses. *J. Environ. Radioact.* **162–163***,* 14–22, DOI: https://doi.org/10.1016/j.jenvrad.2016.05.004 (2016).
19. Steinhauser, G. et al. Post-accident sporadic release of airborne radionuclides from the Fukushima Daiichi Nuclear Power Plant Site. *Environ. Sci. Technol.* **49***,* 14038–14035, DOI: https://doi.org/10.1021/acs.est.5b03155 (2015).
20. Buesseler, K. Michio, A. & Fukasawa, M. Impacts of the Fukushima Nuclear Power Plants on marine radioactivity. *Environ. Sci. Technol.* **45**, 9931–9935, DOI: https://doi.org/10.1021/es202816c (2011).
21. Aoyama, M. et al. ^134^Cs and ^137^Cs in the North Pacific Ocean derived from the March 2011 TEPCO Fukushima Dai‑ichi Nuclear Power Plant accident, Japan. Part two: estimation of ^134^Cs and ^137^Cs inventories in the North Pacific Ocean. *J. Oceanogr.* **72***,* 67–76, DOI: https://doi.org/10.1007/s10872-015-0332-2 (2016).
22. Katata, G. Ota, M. Terada, H., Chino, M. & Nagai, H. Atmospheric discharge and dispersion of radionuclides during the Fukushima Dai-ichi Nuclear Power Plant accident. Part I: Source term estimation and local-scale atmospheric dispersion in early phase of the accident. *J. Environ. Radioact.* **109**, 103–113, DOI: https://doi.org/10.1016/j.jenvrad.2012.02.006 (2012).
23. Katata, G. et al. Detailed source term estimation of the atmospheric release for the Fukushima Daiichi Nuclear Power Station accident by coupling simulations of an atmospheric dispersion model with an improved deposition scheme and oceanic dispersion model. *Atmos. Chem. Phys.* **15**, 1029–1070, DOI: https://doi.org/10.5194/acp-15-1029-2015 (2015).
24. Kajino, M. et al. Deposition and dispersion of radio-cesium released due to the Fukushima Daiichi Nuclear Accident: sensitivity to meteorological models and physical modules. *J. Geophys. Res-Atmos.* **124**, 1823–1845, DOI: https://doi.org/10.1029/2018JD028998 (2019).
25. Saito, K. et al. Detailed deposition density maps constructed by large-scale soil sampling for gamma-ray emitting radioactive nuclides from the Fukushima Dai-ichi Nuclear Power Plant accident. *J. Environ. Radioact.* **139**, 308–319, DOI: https://doi.org/10.1016/j.jenvrad.2014.02.014 (2015).
26. Adachi, K. Kajino, M. Zaizen, Y. & Igarashi, Y. Emission of spherical cesium-bearing particles from an early stage of the Fukushima nuclear accident. *Sci. Rep.* **3**, 2554, DOI: https://doi.org/10.1038/srep02554 (2013).
27. Satou, Y. et al. Analysis of two forms of radioactive particles emitted during the early stages of the Fukushima Dai-ichi Nuclear Power Station accident. *Geochem. J.* **52**, 137–143, DOI: https://doi.org/10.2343/geochemj.2.0514 (2018).
28. Kaneyasu, N. Ohashi, H. Suzuki, F. Okuda, T. & Ikemori, F. Sulfate aerosol as a potential transport medium of radiocesium from the Fukushima nuclear accident. *Environ. Sci. Technol.* **46** (11), 5720−5726, DOI: https://doi.org/10.1021/es204667h (2012).
29. Igarashi, Y., Kajino, M., Zaizen, Y., Adachi, K. & Miakami, M. Atmospheric radioactivity over Tsukuba, Japan: a summary of three years of observations after the FDNPP accident. *Prog. Earth Planet. Sci.* **2**, 44, DOI: https://doi.org/10.1186/s40645-015-0066-1 (2015).
30. Kitayama, K., Ohse, K., Shima, N., Kawatsu, K. & Ysukada, H. Regression model analysis of the decreasing trend of cesium-137 concentration in the atmosphere since the Fukushima accident. *J. Environ. Radioact.* **164**, 151–157, DOI: https://doi.org/10.1016/j.jenvrad.2016.07.015 (2016).
31. Ochiai, S. et al. Temporal variation of post-accident atmospheric ^137^Cs in an evacuated area of Fukushima Prefecture: Size-dependent behaviors of ^137^Cs-bearing particles. *J. Environ. Radioact.* **165**, 131–139, DOI: https://doi.org/10.1016/j.jenvrad.2016.09.014 (2016).
32. Zaunbrecher, L., K., Cygan, R., T. & Elliott, W., C. Molecular models of cesium and rubidium adsorption on weathered micaceous minerals. *J. Phys. Chem.* **119**, 5691–5700, DOI: https://doi.org/10.1021/jp512824k (2015)
33. Ishizuka, M. et al. Use of a size-resolved 1-D resuspension scheme to evaluate resuspended radioactive material associated with mineral dust particles from the ground surface. *J. Environ. Radioact.* **166**, 436–448, DOI: https://doi.org/10.1016/j.jenvrad.2015.12.023 (2017).
34. Kajino, M. et al. Long-term assessment of airborne radiocesium after the Fukushima nuclear accident: re-suspension from bare soil and forest ecosystems. *Atmos. Chem. Phys.* **16**, 13149–13172, DOI: https://doi.org/10.5194/acp-16-13149-2016 (2016).
35. Igarashi, Y. et al. Fungal spore involvement in the resuspension of radiocaesium in summer. *Sci. Rep.* **9**, 1954, DOI: https://doi.org/10.1038/s41598-018-37698-x (2019).
36. Kinase, K. et al. The seasonal variations of atmospheric ^134,137^Cs activity and possible host particles for their resuspension in the contaminated areas of Tsushima and Yamakiya, Fukushima, Japan. *Prog. Earth Planet. Sci.* **5***,* 12, DOI: https://doi.org/10.1186/s40645-018-0171-z (2018).
37. Unterweger, M. P. Half-life measurements at the National Institute of Standards and Technology. *Appl. Radiat. Isotopes.* **56**, 125–130, DOI: https://doi.org/10.1016/S0969-8043(01)00177-4 (2002).
38. MacMahon, D., Pearce, A. & Harris P. Convergence of techniques for the evaluation of discrepant data. *Appl. Radiat. Isotopes.* **60** (2–4), 275–281, DOI: https://doi.org/10.1016/j.apradiso.2003.11.028 (2004).
39. Steinhauser, G., Brandl, A., & Johnson, T., E. Comparison of the Chernobyl and Fukushima nuclear accidents: A review of the environmental impacts. *Sci. Total Environ.*, **470-471**, 800-817, DOI: http://dx.doi.org/10.1016/j.scitotenv.2013.10.029 (2014).
40. Aoyama, M. Evidence of stratospheric fallout of caesium isotopes from the Chernobyl accident. *Geophys. Res. Lett.* **15**, 4, 327–330, DOI: https://doi.org/10.1029/GL015i004p00327 (1988).
41. Aoyama, M., Hirose, K. & Sugimura, Y. The temporal variation of stratospheric fallout derived from the Chernobyl accident. *J. Environ. Radioact.* **13** (2), 103–115, DOI: https://doi.org/10.1016/0265-931X(91)90053-I (1991).
42. Sekiyama, T. T., Kunii, M., Kajino, M. & Shimbori, T. Horizontal Resolution Dependence of Atmospheric Simulations of the Fukushima Nuclear Accident Using 15-km, 3-km, and 500-m Grid Models. *J. Meteorol. Soc. JPN.* **93** (1), 49–64, DOI: https://doi.org/10.2151/jmsj.2015-002 (2015).
43. Sekiyama, T. T. & Kajino, M. Reproducibility of surface wind and tracer transport simulations over complex terrain using 5-, 3-, and 1-km grid models. *J. Appl. Meteorol. Climatol.* **59** (5), 937–952, DOI: https://doi.org/10.1175/JAMC-D-19-0241.1 (2020).
44. Tsuruta, H. Oura, Y. Ebihara, M. Ohara, T. & Nakajima, T. First retrieval of hourly atmospheric radionuclides just after the Fukushima accident by analyzing filter-tapes of operational air pollution monitoring stations. *Sci. Rep.* **4**, 6717 DOI: https://doi.org/10.1038/srep06717 (2014).
45. Igarashi, Y., Aoyama, M., Hirose, K., Povenic, P. & Yabuki, S. What anthropogenic radionuclides (^90^Sr and ^137^Cs) in atmospheric deposition, surface soils and aeolian dusts suggest for dust transport over Japan. *Water, Air, Soil Pollut.* **5**, 51–69, DOI: https://doi.org/10.1007/s11267-005-0726-z (2005).
46. Igarashi, Y., Fujiwara, H.& Jugder, D. Change of the Asian dust source region deduced from the composition of anthropogenic radionuclides in surface soil in Mongolia. *Atmos. Chem. Phys.* **11**, 7069–7080, DOI: https://doi.org/10.5194/acp-11-7069-2011 (2011).
47. Houle, D. Quimet, R. Paquin, R. & Laflamme, J-G. Interactions of atmospheric deposition with a mixed hardwood and a coniferous forest canopy at the Lake Clair Watershed (Duchesnay, Quebec). *Can. J. For. Res.* **29**, 1944–1957, DOI: https://doi.org/10.1139/x99-212 (1999).
48. Su, L., Zhao, C., Xu, W. & Xie, Z. Hydrochemical fluxes in bulk precipitation, throughfall, and stemflow in a mixed evergreen and deciduous broadleaved forest. *Forest*. **10**, 507, DOI: https://doi.org/10.3390/f10060507 (2019).
49. Uehara, Y. et al. Atmospheric deposition and interactions with Pinus pumila regal canopy on Mount Tateyama in the northern Japanese Alps. *Arctic and Alpine Research* **47**, 2, 389–399, DOI: https://doi.org/10.1657/AAAR0013-126 (2015).
50. Katsuragi, Y., Hirose, K. & Sugimura, Y. A study of Plutonium fallout in Japan. *Pap. Meteorol. Geophys.* **33** (2), 85–93, DOI: https://doi.org/10.2467/mripapers.33.85 (1982).
51. Otsuji-Hatori, M., Igarashi, Y. & Hirose, K. Preparation of a reference fallout material for activity measurements. *J. Environ. Radioact.* **3** (2), 143–155, DOI: https://doi.org/10.1016/0265-931X(95)00048-F (1996).

**Acknowledgments**

The authors thank Kenzo Hama, Keiko Kamioka and Minami Ide-Tanimoto (ATOX Co. Ltd.) for the samplings and measurements and Kayo Yanagida, Kyoko Kaneba and Joseph Ching (MRI) for sampling atmospheric deposition at site A.

This study was financially supported by the former Ministry of Education, Culture, Sports, Science and Technology and the current Nuclear Regulation Authority (Japanese Radioactivity Survey) and the Environmental Restoration and Conservation agency (grant number: 1-1801).

Author Contributions

TK, YI and YZ collected samples. TK and MK analyzed the data. YI, TS, MK, and YZ managed the project. TK and KA wrote this paper, and all authors contributed to discussions.

**Competing interests**

The authors declare no competing financial interests.
